# Impact of Medicaid expansion on young adult firearm and motor vehicle crash trauma patients

**DOI:** 10.1016/j.sopen.2022.01.004

**Published:** 2022-02-01

**Authors:** Michael R. Ross, Philip M. Hurst, Lindsey Asti, Jennifer N Cooper

**Affiliations:** a The Ohio State University College of Medicine, 370 W 9th Ave, Columbus, OH 43210, United States; b Medical Student Research Program, The Ohio State University College of Medicine, 370 W 9th Ave, Columbus, OH 43210, United States; c Center for Surgical Outcomes Research, Abigail Wexner Research Institute at Nationwide Children's Hospital, 700 Children's Dr, Columbus, OH 43205, United States; d Center for Child Health Equity and Outcomes Research, Abigail Wexner Research Institute at Nationwide Children's Hospital, 700 Children's Dr, Columbus, OH 43205, United States; e Department of Pediatrics, College of Medicine, The Ohio State University, 370 W 9th Ave, Columbus, OH 43210, United States; f Division of Epidemiology, College of Public Health, The Ohio State University, 1841 Neil Ave Columbus, OH 43210, United States

## Abstract

**Background:**

The Affordable Care Act Medicaid expansion has increased insurance coverage and reduced some disparities in care and outcomes among trauma patients, but its impact on subsets of trauma patients with particular mechanisms of injury are unclear. This study evaluated the association of the Affordable Care Act Medicaid expansion with insurance coverage, trauma care, and outcomes among young adults hospitalized for firearm- or motor vehicle crash–related injuries.

**Materials and Methods:**

We used statewide hospital discharge data from 5 Medicaid expansion and 5 nonexpansion states to compare changes in insurance coverage and outcomes among firearm and motor vehicle crash trauma patients aged 19–44 from before (2011–2013) to after (2014–2017) Medicaid expansion. We examined difference in differences overall, by race/ethnicity, and by zip-code-level median income quartile.

**Results:**

Medicaid expansion was associated with a decrease in the proportion of young adult motor vehicle crash and firearm trauma patients who were uninsured (motor vehicle crash: difference in differences − 12.7 percentage points, P < .001; firearm: difference in differences − 30.7 percentage points, P < .001). Medicaid expansion was also associated with increases in the percentage of patients discharged to any rehabilitation (motor vehicle crash: difference in differences 1.78 percentage points, P = .001; firearm: difference in differences 2.07 percentage points, P = .02) and inpatient rehabilitation (motor vehicle crash: difference in differences 1.21 percentage points, P = .001; firearm: difference in differences 1.58 percentage points, P = .002). Among patients with firearm injuries, Medicaid expansion was associated with a reduction in in-hospital mortality (difference in differences − 1.55 percentage points, P = .002).

**Conclusion:**

In its first 4 years, the Affordable Care Act Medicaid expansion increased insurance coverage and access to rehabilitation among young adults hospitalized for firearm- or motor vehicle crash–related injuries while reducing inpatient mortality among firearm trauma patients.

## INTRODUCTION

Traumatic injuries are the leading cause of death and disability among adults under age 45 [1]. They also result in billions of dollars in medical and work loss related costs every year [2]. Previous research has shown that patients of low socioeconomic status (SES) and racial/ethnic minorities are more likely to experience poor quality trauma care, worse outcomes, and a lack of access to rehabilitative care after traumatic injury [3,4]. Prior to the implementation of the Affordable Care Act's (ACA's) insurance coverage expansions in 2014, more than 30% of young adult patients were uninsured, and that percentage was much higher among low SES and racial/ethnic minority patients [5]. Numerous studies prior to the ACA also showed that uninsured trauma patients were more likely to die in the hospital and less likely to receive rehabilitative care than insured trauma patients even after accounting for patient comorbidities and injury characteristics [3,4,[6], [7], [8]].

Motor vehicle crashes (MVCs) and firearms are among the most common mechanisms of traumatic injury in the United States [9]. Sehgal documented that Americans have a 0.92% and 0.93% lifetime risk of dying from an MVC or firearm injury, respectively [10]. Although the population-level mortality rates associated with these 2 mechanisms of injury are quite similar, the distributions of sociodemographic groups affected, the intentionality of these injuries, and the likelihood of these patients to be uninsured differ markedly [11,12]. MVCs are almost exclusively accidental, whereas firearm injuries are often intentional [9]. Nevertheless, many of the aforementioned racial/ethnic and socioeconomic disparities that are seen in the overall trauma population exist among both firearm and MVC trauma patients [8,[13], [14], [15], [16], [17], [18], [19], [20]].

The ACA featured a number of provisions intended to expand health insurance coverage and improve the health of Americans [21]. Of all the ACA provisions, Medicaid expansion has had the greatest impact on reducing the uninsured rate among nonelderly adults [[22], [23], [24]]. One national survey found that the percentage of uninsured Americans aged 18–64 declined from 22.3% in 2010 to 13.3% in 2018, with the most dramatic drops observed in Medicaid expansion states [25]. Among trauma patients specifically, several studies have described the effects of Medicaid expansion on insurance coverage, trauma care, and outcomes. These studies have found that Medicaid expansion resulted in a greater than 15 percentage point decrease in the uninsured rate among young adult trauma patients overall in 2014 and a continued smaller decrease in the years thereafter [[26], [27], [28], [29]]. Medicaid expansion also significantly decreased insurance coverage disparities between non-Hispanic black and non-Hispanic white trauma patients and between patients residing in lower- versus higher-income zip codes [26,30]. Furthermore, Medicaid expansion increased access to rehabilitation and decreased socioeconomic disparities in access to rehabilitation [[27], [28], [29], [30]]. We also found that, among all young adult trauma patients, Medicaid expansion, in its first 4 years of implementation, did not reduce in-hospital mortality or unplanned readmissions overall but did reduce the disparity in in-hospital mortality between black and white trauma patients [26].

The young adult trauma population is very heterogeneous with regard to types and mechanisms of injury, but the effects of Medicaid expansion on patients with specific mechanisms of injury, such as MVC- and firearm-related trauma, are unclear. The objective of this study was to investigate the impact of the ACA Medicaid expansion, in its first 4 years, on insurance coverage, trauma outcomes, and access to rehabilitation among young adult trauma patients with firearm- or MVC-related injuries. We examined the impact of the ACA Medicaid expansion in these groups overall and across subgroups defined by patient race/ethnicity and community income level.

## MATERIALS AND METHODS

### Data Sources

This study used the state inpatient databases (SIDs) of 5 Southern and Midwestern states that implemented the ACA Medicaid expansion in January 2014 (Arkansas, Iowa, Illinois, Kentucky, and Maryland) and 5 Southern and Midwestern states that had not yet adopted the ACA Medicaid expansion as of December 2017 (Florida, Georgia, Kansas, Missouri, and North Carolina). The SIDs of these states were obtained from the Agency for Healthcare Research and Quality's (AHRQ's) Healthcare Cost and Utilization Projection central distributor or from state departments of health. These 5 Medicaid expansion states and 5 non-Medicaid expansion states were selected because of their similarity in geographic, sociodemographic, and trauma system characteristics (Supplemental Table S1). In addition, these 10 states also (1) have a young adult population composed of at least 10% black or Hispanic individuals, (2) had not implemented a state-level policy that expanded insurance coverage to low-income adults without dependents prior to 2014, and (3) had less than 10% missing patient race/ethnicity and zip-code-level median household income quartile or patient residential zip code data. We elected to focus on Hispanic, non-Hispanic black, and non-Hispanic white individuals because these are the largest racial/ethnic groups in the United States and most previous studies of racial/ethnic disparities in trauma care and outcomes have focused on these groups. Each SID includes all inpatient discharge records from all community hospitals in the state. Data are derived from hospital discharge summaries and billing records. The investigators completed data use agreements with AHRQ, the Illinois Department of Public Health, and the Missouri Department of Health and Senior Services. This study was approved by the Institutional Review Board of the Illinois Department of Public Health and by our local Institutional Review Board with a waiver of informed consent.

### Study Population

We included patients aged 19–44 years who were hospitalized at an acute care community hospital for traumatic firearm- or MVC-related injuries and who were discharged in 2011–2017. To identify hospitalizations for traumatic injury, we followed the National Trauma Data Standard [31]. Patients with an International Classification of Diseases (ICD)-9-CM external cause of injury code indicating that their injury was firearm-related (E922(.0–.9) or E955(.0–.4)) or MVC-related ([E810–E819] (.0–.9) or E958.5) were included [32]. Patients with an ICD-10-CM external cause of injury code indicating firearm injury (W32, W33, W34.00, W34.09, W34.10, W34.19, X72, X73, X74.8, X74.9, X93, X94, X95.8, X95.9, Y22, Y23, Y24.8, Y24.9, Y35.00–Y35.03, Y35.09, Y36.42, Y36.43, Y36.92, Y37.42, Y37.43, Y37.92 Y38.4) or MVC injury (V01–V89, V91, V93–V99, X81.0, X81.1, X82, X83.0, Y02.0, Y02.1, Y03, Y08.81, Y32, Y36.1, Y37.1, Y38.1) were also included [33]. Patients were also required to be residents of the state in which they were hospitalized. Patients were excluded from the study population if they were transferred to an acute care hospital in another state not included in the study or to an in-state acute care hospital not included in the database. They were also excluded if their admission was elective or if they were missing data on discharge disposition, sex, race/ethnicity, national zip-code-level median household income quartile, or urban/rural residence. Less than 3% of admissions were excluded owing to having missing data on these key factors.

### Covariates and Outcomes

The key outcomes investigated in this study were the type of insurance coverage (uninsured, Medicaid, private insurance, other), in-hospital mortality, discharge to any rehabilitation (inpatient rehabilitation facility or unit, skilled nursing facility or unit, or home health care), and any unplanned readmission or emergency department (ED) visit within 30 days. *Unplanned readmissions* were defined as nonelective readmissions. Unplanned readmissions and return ED visits were examined only in states with patient identifiers (Arkansas, Iowa, Illinois, Maryland, Florida, and Georgia) and only in 2013–2017 owing to patient identifiers being absent in some states in earlier years. The key exposures examined were year of discharge, state Medicaid expansion status, patient race/ethnicity, and patient residential zip-code-level median household income quartile. However, data on other patient factors that could affect outcomes in trauma patients were examined and included in the risk-adjusted outcome models. These factors included the following: patient age, sex, urban/rural residence, injury severity score (ISS), presence of a severe head or neck injury, presence of any chronic condition, presence of traumatic shock, and whether an operative procedure was performed. These factors were included in the multivariable analyses because of their established relationships with one or more of the outcomes of interest [26,[34], [35], [36], [37], [38]]. Patient residential urbanicity/rurality was evaluated using the National Center for Health Statistics' county-level scheme [39]. Injury severity scores were calculated using the ICD Program for Injury Categorization in R statistical software, version 0.1.0 (ICDPIC-R) [40]. The original ICDPIC program was developed for use with ICD-9-CM codes, but ICDPIC-R allows the use of both ICD-9-CM and ICD-10-CM codes, with options to calculate injury severity directly from ICD-10-CM codes (based on diagnosis-specific mortality estimates from the National Trauma Data Bank) or indirectly by first mapping ICD-10-CM codes to ICD-9-CM codes using the Centers for Medicare & Medicaid Services' (CMS') General Equivalence Mapping (GEM) tables. We chose to use the GEM minimum severity method for converting ICD-10-CM codes to ICD-9-CM codes because this strategy resulted in minimal disruption in trends across the coding transition [41]. Hospital characteristics, such as trauma center status and level, were not able to be considered because hospital identifiers that could be used to link the SID to other data at the hospital level were not available for Georgia, Kansas, or Missouri.

### Statistical Analysis

All analyses were stratified by mechanism of injury. Similar to our analyses in the overall young adult trauma patient population [26], in this study, we examined patient characteristics in the overall study cohort and in Medicaid expansion and nonexpansion states before and after the implementation of the ACA Medicaid expansion and open enrollment in January 2014. We summarized these characteristics using frequencies and percentages, medians and interquartile ranges, or means and standard deviations (SDs). We verified the parallel trends assumption required for difference-in-differences (DD) analyses by comparing trends in outcomes in 2011–2013 between expansion and nonexpansion states overall and within each racial/ethnic and zip-code-level income group. We then conducted DD analyses to examine the impact of the ACA Medicaid expansion on insurance coverage and risk-adjusted outcomes. For the DD analyses, we used marginal logistic regression models fit using generalized estimating equations to account for patient clustering within hospitals. The models included time period (before versus after Medicaid expansion and open enrollment), state Medicaid expansion status, and their interaction. Models for insurance coverage did not contain additional covariates, but all other outcome models included patient age, sex, urban/rural residence, ISS category (mild or moderate: ISS ≤ 15, severe: 16 ≤ ISS ≤ 24, extremely severe: ISS ≥ 25), whether a severe head or neck injury (head/neck abbreviated injury scale (AIS) ≥ 3) was present, and whether the patient had any chronic conditions. Models for in-hospital mortality additionally included a binary variable for whether the patient had experienced traumatic shock. Models for unplanned readmissions and return ED visits also included a binary variable for whether a surgical procedure had been performed, as this is strongly associated with readmission risk [[42], [43], [44]].

To evaluate the impact of the ACA Medicaid expansion on racial/ethnic and socioeconomic disparities in insurance coverage and risk-adjusted outcomes, we performed difference-in-difference-difference (DDD) analyses using an analogous modeling approach. These models included the same covariates as described above but additionally included second-order interactions between time period, state Medicaid expansion status, and either patient race/ethnicity or the median national household income quartile of the patient's residential zip code. All estimates of differences between Medicaid expansion and nonexpansion states in changes in risk-adjusted outcomes from before to after Medicaid expansion and open enrollment were derived using marginal standardization either for the overall study cohort (in DD analyses) or within each sociodemographic group of interest (in DDD analyses) [45,46]. We also conducted analyses in which DD and DDD estimates were derived for each year after the implementation of the ACA Medicaid expansion and open enrollment (2014–2017) as compared to 2011–2013.

We repeated our analyses of the discharge to rehabilitation outcomes after restricting the study population to patients who had 1 or more of the presumptive diagnosis codes defined by CMS as a requirement for financial support of inpatient rehabilitation facilities (CMS-funded inpatient rehabilitation facilities must support a case mix of patients comprising 60% or more of such codes, ie, the 60% rule) [47]. All statistical analyses were performed using SAS Enterprise Guide version 8.1 (SAS Institute Inc, Cary, NC).

## RESULTS

### Patient Characteristics

Our study population was composed of 34,946 firearm trauma patients and 129,439 MVC trauma patients. Within this study population, 12,367 (35.4%) firearm trauma patients and 37,256 (28.8%) MVC trauma patients were from states that expanded Medicaid under the ACA. The patient population was predominantly male (90.4% for firearm, 67.3% for MVC) with a mean age of 27.8 (SD 6.8) years among firearm trauma patients and 29.8 (SD 7.5) years among MVC trauma patients. Sociodemographic characteristics of the study population varied between the 2 groups. The MVC cohort was composed mostly of non-Hispanic white patients (60.2%). The firearm trauma patient cohort was made up of 21.0% non-Hispanic white, 67.2% non-Hispanic black, and 8.3% Hispanic patients. A majority (57.4%) of the firearm trauma study population came from the lowest income quartile, whereas the MVC trauma study population was more evenly distributed socioeconomically. In both groups, patients in Medicaid expansion states were more likely to live in higher-income zip codes than patients from nonexpansion states. (See Table 1.)

**Table 1:** Characteristics and outcomes of young adult firearm and motor vehicle crash (MVC) trauma patients by time period and state Medicaid expansion status

	*2011–2013*				*2014–2017*			
	*Firearm trauma patients*		*MVC trauma patients*		*Firearm trauma patients*		*MVC trauma patients*	
	*Expansion states (*n *= 4,973)*	*Nonexpansion states (*n *= 8,789)*	*Expansionstates (*n *= 17,190)*	*Nonexpansion states (*n *= 38,931)*	*Expansion states (*n *= 7,394)*	*Nonexpansion states (*n *= 13,790)*	*Expansion states (*n *= 20,066)*	*Nonexpansion states (*n *= 53,252)*
	n *(%)*	n *(%)*	n *(%)*	n *(%)*	n *(%)*	n *(%)*	n *(%)*	n *(%)*
*Patient characteristics*								
Age, y [mean (SD)]	27.3 (6.7)	28.0 (6.9)	29.8 (7.6)	29.8 (7.6)	27.5 (6.6)	28.1 (6.8)	30.0 (7.4)	29.8 (7.4)
Sex								
Male	4,574 (92.0)	7,927 (90.2)	11,631 (67.7)	26,082 (67.0)	6,701 (90.6)	12,393 (89.9)	13,348 (66.5)	35,998 (67.6)
Female	399 (8.0)	862 (9.8)	5,559 (32.3)	12,849 (33.0)	693 (9.4)	1,397 (10.1)	6,718 (33.5)	17,254 (32.4)
Race/ethnicity								
Non-Hispanic white	865 (17.4)	2,133 (24.3)	11,098 (64.6)	24,122 (62.0)	1,285 (17.4)	3,060 (22.2)	12,543 (62.5)	30,186 (56.7)
Non-Hispanic black	3,505 (70.5)	5,657 (64.4)	3,561 (20.7)	8,214 (21.1)	5,127 (69.3)	9,199 (66.7)	4,288 (21.4)	13,176 (24.7)
Hispanic (any race)	479 (9.6)	715 (8.1)	1,626 (9.5)	5,069 (13.0)	585 (7.9)	1,110 (8.0)	2,112 (10.5)	7,726 (14.5)
Other	124 (2.5)	284 (3.2)	905 (5.3)	1,526 (3.9)	397 (5.4)	421 (3.1)	1,123 (5.6)	2,164 (4.1)
Zip-code-level median household income quartile								
Quartile 1 (lowest)	2,881 (57.9)	4,663 (53.1)	5,066 (29.5)	14,162 (36.4)	4,354 (58.9)	8,153 (59.1)	6,527 (32.5)	22,707 (42.6)
Quartile 2	882 (17.7)	2,380 (27.1)	3,286 (19.1)	12,150 (31.2)	1,275 (17.2)	3,719 (27.0)	4,247 (21.2)	17,126 (32.2)
Quartile 3	785 (15.8)	1,338 (15.2)	4,324 (25.2)	8,929 (22.9)	1,184 (16.0)	1,441 (10.4)	4,898 (24.4)	9,625 (18.1)
Quartile 4 (highest)	425 (8.5)	408 (4.6)	4,514 (26.3)	3,690 (9.5)	581 (7.9)	477 (3.5)	4,394 (21.9)	3,794 (7.1)
Primary payer (*n* = 34,812 firearm; *n* = 128,998 MVC)								
Medicare	94 (1.9)	191 (2.2)	290 (1.7)	723 (1.9)	132 (1.8)	272 (2.0)	346 (1.7)	768 (1.4)
Medicaid	1,154 (23.2)	1,545 (17.7)	2,310 (13.5)	4,231 (10.9)	4,465 (60.9)	2,609 (19.0)	6,106 (30.7)	5,746 (10.8)
Private insurance	1,148 (23.1)	1,724 (19.7)	9,515 (55.6)	20,543 (52.9)	1,591 (21.7)	2,594 (18.9)	9,964 (50.1)	29,000 (54.5)
Self-pay	1,872 (37.7)	3,618 (41.3)	3,441 (20.1)	8,100 (20.9)	835 (11.4)	5,796 (42.1)	1,642 (8.2)	10,805 (20.3)
No charge	167 (3.4)	463 (5.3)	177 (1.0)	770 (2.0)	25 (0.3)	815 (5.9)	28 (0.1)	1,306 (2.5)
Other	531 (10.7)	1,210 (13.8)	1,390 (8.1)	4,439 (11.4)	287 (3.9)	1,674 (12.2)	1,819 (9.1)	5,539 (10.4)
Urban/rural residence								
Large central metropolitan (> 1 million population)	2,952 (59.4)	3,003 (34.2)	3,833 (22.3)	9,233 (23.7)	4,312 (58.3)	5,013 (36.4)	4,923 (24.5)	14,206 (26.7)
Large fringe metropolitan (> 1 million population)	614 (12.3)	2,129 (24.2)	5,079 (29.5)	10,823 (27.8)	868 (11.7)	3,691 (26.8)	4,852 (24.2)	15,944 (29.9)
Medium metropolitan (250,000–999,999 population)	660 (13.3)	1,949 (22.2)	2,765 (16.1)	8,825 (22.7)	1,052 (14.2)	2,612 (18.9)	3,521 (17.5)	11,768 (22.1)
Small metropolitan (50,000–249,999 population)	323 (6.5)	634 (7.2)	1,630 (9.5)	3,135 (8.1)	528 (7.1)	1,057 (7.7)	2,008 (10.0)	4,299 (8.1)
Micropolitan	198 (4.0)	752 (8.6)	2,018 (11.7)	4,176 (10.7)	335 (4.5)	943 (6.8)	2,303 (11.5)	4,029 (7.6)
Noncore	226 (4.5)	322 (3.7)	1,865 (10.8)	2,739 (7.0)	299 (4.0)	474 (3.4)	2,459 (12.3)	3,006 (5.6)
No. of chronic conditions								
0	1,753 (35.3)	3,307 (37.6)	5,085 (29.6)	12,867 (33.1)	2,213 (29.9)	4,241 (30.8)	4,984 (24.8)	15,001 (28.2)
1	1,341 (27.0)	2,365 (26.9)	4,802 (27.9)	10,583 (27.2)	2,055 (27.8)	3,910 (28.4)	5,284 (26.3)	14,619 (27.5)
2	878 (17.7)	1,464 (16.7)	3,243 (18.9)	7,033 (18.1)	1,328 (18.0)	2,466 (17.9)	3,925 (19.6)	10,103 (19.0)
3	505 (10.2)	806 (9.2)	1,906 (11.1)	4,129 (10.6)	807 (10.9)	1,462 (10.6)	2,429 (12.1)	5,991 (11.3)
> 3	496 (10.0)	847 (9.6)	2,154 (12.5)	4,319 (11.1)	991 (13.4)	1,711 (12.4)	3,444 (17.2)	7,538 (14.2)
Operative procedure	3,271 (65.8)	6,033 (68.6)	8,409 (48.9)	22,013 (56.5)	5,218 (70.6)	9,628 (69.8)	11,472 (57.2)	31,186 (58.6)
Traumatic shock	308 (6.2)	639 (7.3)	387 (2.3)	1,040 (2.7)	649 (8.8)	1,259 (9.1)	639 (3.2)	1,747 (3.3)
ISS (*n* = 34,946 firearm; *n* = 129,437 MVC)								
Mild/moderate (0–15)	3,742 (75.2)	6,756 (76.9)	12,629 (73.5)	27,930 (71.7)	5,596 (75.7)	10,944 (79.4)	13,608 (67.8)	37,187 (69.8)
Severe (16–24)	666 (13.4)	1,224 (13.9)	3,175 (18.5)	7,765 (19.9)	1,020 (13.8)	1,581 (11.5)	4,252 (21.2)	10,854 (20.4)
Extremely severe (25–75)	565 (11.4)	809 (9.2)	1,384 (8.1)	3,236 (8.3)	778 (10.5)	1,265 (9.2)	2,206 (11.0)	5,211 (9.8)
Severe head or neck injury (AIS ≥ 3)	618 (12.4)	1,018 (11.6)	3,509 (20.4)	8,087 (20.8)	905 (12.2)	1,432 (10.4)	4,227 (21.1)	10,448 (19.6)
Injury intent								
Undetermined/unintentional	1,282 (25.8)	2,397 (27.3)	17,190 (100.0)	38,931 (100.0)	1,995 (27.0)	5,510 (40.0)	19,998 (99.7)	53,111 (99.7)
Self-harm	209 (4.2)	531 (6.0)	0	0	292 (3.9)	682 (4.9)	41 (0.2)	73 (0.1)
Assault	3,426 (68.9)	5,694 (64.8)	0	0	5,027 (68.0)	7,451 (54.0)	27 (0.1)	68 (0.1)

*Outcomes*								
In-hospital mortality	420 (8.4)	563 (6.4)	364 (2.1)	891 (2.3)	509 (6.9)	827 (6.0)	462 (2.3)	1,241 (2.3)
Discharged to any rehabilitation⁎	528 (15.1)	1,086 (13.2)	2,492 (20.8)	7,746 (20.4)	892 (16.2)	1,600 (12.3)	3,875 (24.5)	11,257 (21.6)
Discharged to inpatient rehabilitation⁎	155 (4.4)	382 (4.6)	1,183 (9.9)	3,633 (9.6)	330 (6.0)	618 (4.8)	2,040 (12.9)	5,611 (10.8)
Discharged to a skilled nursing facility⁎	27 (0.8)	65 (0.8)	227 (1.9)	773 (2.0)	44 (.8)	77 (.6)	435 (2.8)	1,042 (2.0)
Discharged to home health care⁎	346 (9.9)	639 (7.8)	10,893 (91.0)	34,700 (91.2)	518 (9.4)	905 (7.0)	1,400 (8.9)	4,604 (8.9)
30-d unplanned readmission^†^	113 (9.3)	149 (8.9)	313 (7.8)	523 (7.1)	656 (10.1)	683 (9.3)	1,297 (8.2)	2,319 (7.5)
30-d return ED visit†	252 (20.8)	391 (23.4)	598 (14.8)	1,313 (17.9)	1,588 (24.5)	1,827 (24.9)	2,476 (15.7)	5,758 (18.6)

⁎ Missing for Maryland: *n* = 30,210 firearm; *n* = 117,816 MVC.

† For 2013–2017 only; missing for Kansas, Kentucky, Missouri, and North Carolina: *n* = 16,719 firearm, *n* = 58,160 MVC.

### Effects of the ACA Medicaid Expansion on Insurance Coverage

**Fig 1:**
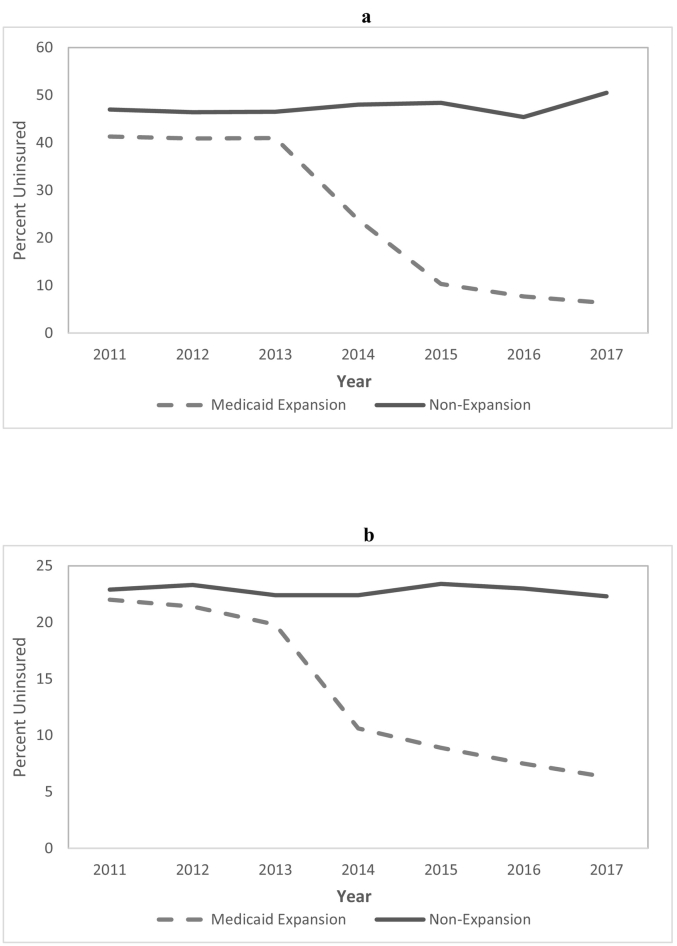
Proportion of young adult (a) firearm trauma patients and (b) motor vehicle crash trauma patients uninsured over time, by state Medicaid expansion status.

**Fig 2:**
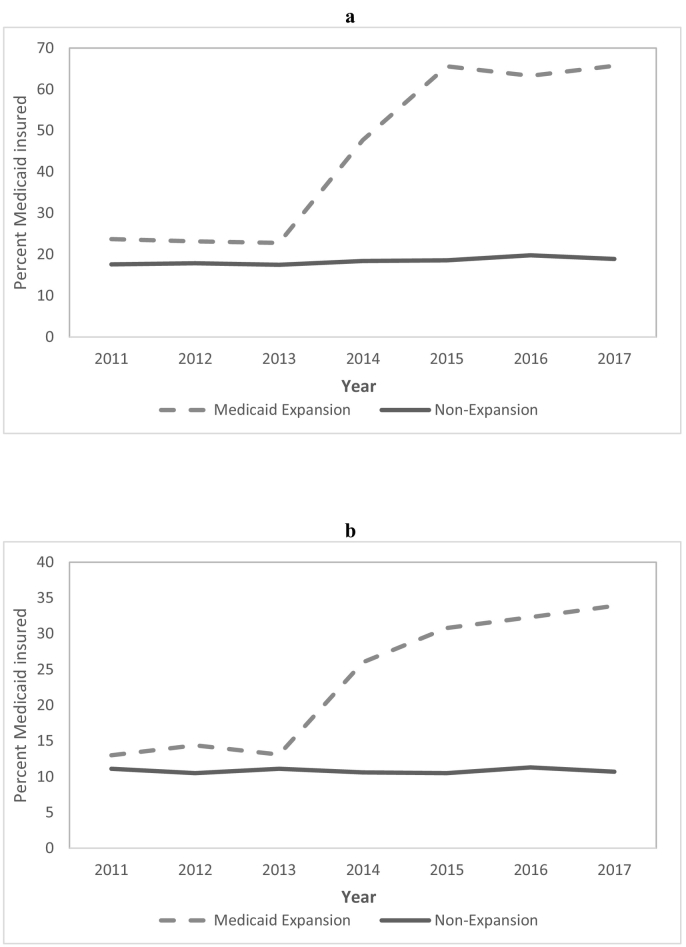
Proportion of young adult (a) firearm trauma patients and (b) motor vehicle crash trauma patients covered by Medicaid over time, by state Medicaid expansion status.

In the first 4 years after the implementation of the ACA Medicaid expansion, the uninsured rate dropped by 12.7 and 29.4 percentage points in the selected expansion states among MVC and firearm trauma patients, respectively (Table 2). Meanwhile, the uninsured rate in the nonexpansion states decreased by only 0.1 percentage point among MVC trauma patients and increased by 1.4 percentage points among firearm trauma patients. Thus, we found that the proportion of young adult MVC and firearm trauma patients who were uninsured decreased significantly more in the selected Medicaid expansion than nonexpansion states (MVC: DD − 12.66, 95% CI − 13.57 to − 11.76, *P* < .001; firearm: DD − 30.74, 95% CI − 32.80 to − 28.69, *P* < .001). The greatest reductions in the uninsured rates in the expansion states among MVC and firearm trauma patients occurred in the first 2 years after Medicaid expansion (Fig 1). The proportion of patients covered by Medicaid increased in expansion states by 37.7 and 17.2 percentage points in the firearm and MVC trauma populations, respectively. In comparison, Medicaid coverage rates increased by just 1.3 percentage points in the firearm trauma population and declined by 0.1 percentage point in the MVC study population in the nonexpansion states. As a result, the proportion of patients covered by Medicaid increased significantly more in Medicaid expansion states than in nonexpansion states for both trauma mechanisms (MVC: DD 17.28, 95% CI 16.36 to 18.20, *P* < .001; firearm: DD 36.33, 95% CI 34.41 to 38.25, *P* < .001). The greatest increases in the Medicaid coverage rates in expansion states for both groups occurred in the first 2 years after Medicaid expansion (Fig 2). In the MVC cohort, there was a significant reduction in private insurance coverage (− 5.5 percentage points) in the expansion states, whereas there was a 1.6 percentage point increase in the nonexpansion states (DD − 7.12, 95% CI − 8.33 to − 5.91, *P* < .001). Such changes in private insurance coverage were not observed among firearm trauma patients.

There were several differences by patient race/ethnicity and community income level in the impact of Medicaid expansion on insurance coverage (Table 3, Table 4). Among firearm trauma patients, Hispanic patients experienced a smaller Medicaid expansion–associated decrease in their uninsured rate than white patients. In contrast, among MVC trauma patients, black patients experienced a greater Medicaid expansion–associated decrease in their uninsured rate than white patients. Among firearm trauma patients, there was no difference by community income quartile in the impact of Medicaid expansion on the uninsured rate. However, among MVC trauma patients, reductions in the uninsured rate were larger in low- and moderate-income communities than in the highest-income communities.

### Effects of the ACA Medicaid Expansion on Trauma Outcomes and Access to Rehabilitation

**Fig 3:**
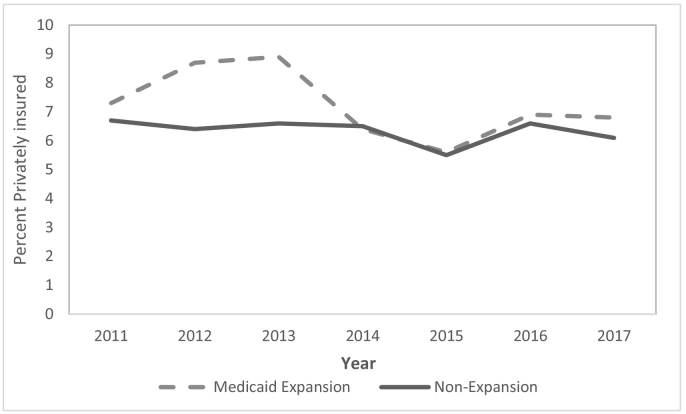
Proportion of young adult firearm trauma patients who died in the hospital over time, by state Medicaid expansion status.

When comparing 2014–2017 with 2011–2013, the proportion of young adult firearm trauma patients who died in the hospital decreased by 1.9 percentage points in the selected expansion states, whereas it decreased by only 0.3 percentage point in the selected nonexpansion states (Table 2). This difference was statistically significant (DD − 1.55, 95% CI − 2.52 to − 0.59, *P* = .002). No association between Medicaid expansion and changes in in-hospital mortality was observed for MVC trauma patients. Although Medicaid expansion was associated with a decrease in in-hospital mortality among firearm trauma patients, this decrease did not vary over time during the postexpansion years (Fig 3).

**Table 2:** Association of the ACA Medicaid expansion with insurance coverage and risk-adjusted outcomes among young adult firearm and MVC trauma patients

	*Expansion states*		*Nonexpansion states*		*Difference-in-differences estimate (95% CI)*	P
	*2011–2013*	*2014–2017*	*2011–2013*	*2014–2017*		
	*(*n *= 4,973 for firearm;* n *= 17,190 for MVC)*	*(*n *= 7,394 for firearm;* n *= 20,066 for MVC)*	*(*n *= 8,789 for firearm;* n *= 38,931 for MVC)*	*(*n *= 13,790 for firearm;* n *= 53,252 for MVC)*		
Uninsured						
Firearm	41.1	11.7*	46.6	48.0*	− 30.74 (− 32.80 to − 28.69)	<.001
MVC	21.1	8.4*	22.9	22.8	− 12.66 (− 13.57 to − 11.76)	<.001
Medicaid						
Firearm	23.2	60.9*	17.7	19.0*	36.33 (34.41 to 38.25)	<.001
MVC	13.5	30.7*	10.9	10.8	17.28 (16.36 to 18.20)	<.001
Private insurance						
Firearm	23.1	21.7	19.7	18.9	− 0.58 (− 2.42 to − 1.26)	.54
MVC	55.6	50.1*	52.9	54.5*	− 7.12 (− 8.33 to − 5.91)	<.001
In-hospital mortality						
Firearm	8.3	6.4*	6.5	6.2	− 1.55 (− 2.52 to − 0.59)	.002
MVC	2.4	2.1*	2.4	2.2*	− 0.09 (− 0.43 to 0.25)	.61
Discharged to any rehabilitation						
Firearm	15.4	16.6	13.0	12.2	2.07 (0.32 to 3.81)	.02
MVC	21.0	23.6*	20.7	21.6*	1.78 (0.70 to 2.87)	.001
Discharged to inpatient rehabilitation						
Firearm	4.4	6.0*	4.7	4.8	1.58 (0.56 to 2.60)	.002
MVC	9.8	12.0*	9.8	10.8*	1.21 (0.42 to 2.08)	.003
Discharged to a skilled nursing facility						
Firearm	0.7	0.8	0.8	0.6	0.24 (− 0.18 to 0.67)	.26
MVC	2.0	2.7*	2.1	2.0	0.83 (0.43 to 1.23)	<.001
Discharged to home health care						
Firearm	10.4	9.9	7.6	6.9	0.17 (− 1.32 to 1.65)	.83
MVC	9.3	8.9	8.8	8.8	− 0.32 (− 1.10 to 0.46)	.83
30-d unplanned readmission						
Firearm	9.2	10.0	9.2	9.3	0.59 (− 1.75 to 2.93)	.62
MVC	7.9	7.9	7.4	7.5	− 0.08 (− 1.22 to 1.06)	.89
30-d return ED visit						
Firearm	21.2	24.6*	23.5	24.6	2.27 (− 1.12 to 5.67)	.19
MVC	15.5	16.3	17.7	18.2	0.36 (− 1.24 to 1.96)	.66

Risk-adjusted marginal percentages are shown. *P < .05 versus 2011–2013 in the same states.

**Fig 4:**
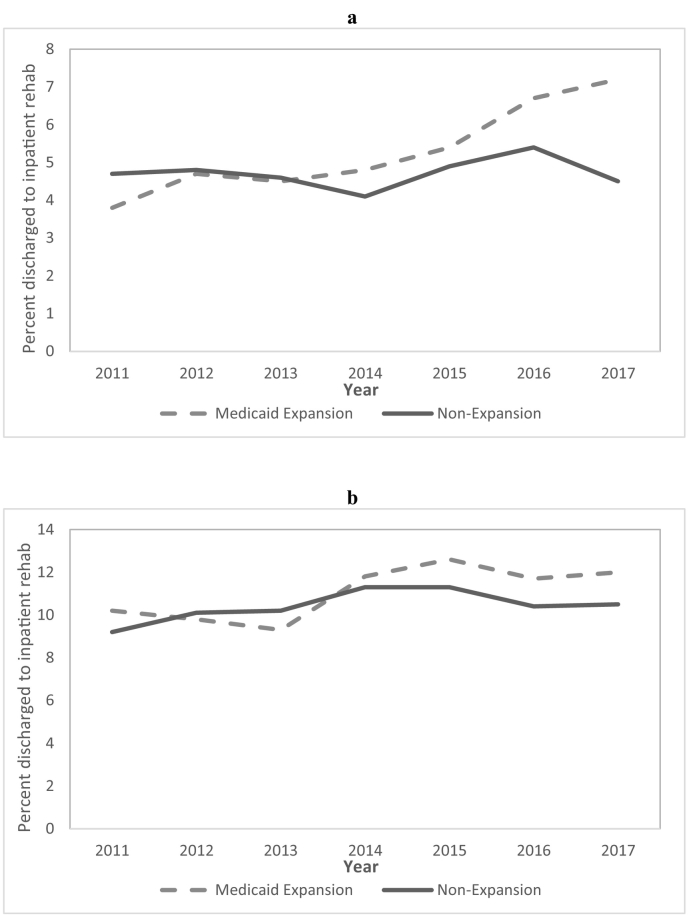
Proportion of young adult (a) firearm trauma patients and (b) motor vehicle crash trauma patients discharged to inpatient rehabilitation over time, by state Medicaid expansion status.

Medicaid expansion was associated with an increase in the proportion of patients discharged to any rehabilitation among both MVC and firearm trauma patients (MVC: DD 1.78, 95% CI 0.70 to 2.87, *P* = .001; firearm: DD 2.07, 95% CI 0.32 to 3.81, *P* = .02) (Table 2). Medicaid expansion was also associated with an increase in the proportion of patients discharged to inpatient rehabilitation among both MVC and firearm trauma patients (MVC: DD 1.21, 95% CI 0.42 to 2.08, *P* = .003; firearm: DD 1.58, 95% CI 0.56 to 2.60, *P* = .002). Conversely, Medicaid expansion was associated with an increase in the proportion of patients discharged to skilled nursing among MVC trauma patients but not among firearm trauma patients. Among the firearm trauma patients, the impact of Medicaid expansion on access to inpatient rehabilitation increased from year to year, whereas no significant trend was observed among MVC trauma patients (Fig 4). Medicaid expansion was not associated with changes in the rates of discharge to home health care, any 30-day unplanned readmission, or any 30-day return ED visit in either the firearm or MVC trauma patient cohorts.

Regarding the differential impact of Medicaid expansion by patient race/ethnicity, black MVC trauma patients relative to white patients saw larger gains in discharge to any rehabilitation and discharge specifically to skilled nursing. Black patients also experienced a greater Medicaid expansion–associated increase in the proportion of patients with a return ED visit within 30 days (Table 3). None of the other trauma outcomes evaluated in MVC trauma patients and none of the outcomes evaluated in firearm trauma patients showed significant racial or ethnic differences in their association with Medicaid expansion.

**Table 3:** Association of the ACA Medicaid expansion with insurance coverage and risk-adjusted outcomes among young adult firearm and MVC trauma patients: results by race/ethnicity

	*Firearm trauma patients*					*MVC trauma patients*				
	*Expansion states*		*Nonexpansion states*		*Difference-in-differences estimate (95% CI)*	*Expansion states*		*Nonexpansion states*		*Difference-in-differences estimate (95% CI)*
	*2011–2013*	*2014–2017*	*2011–2013*	*2014–2017*		*2011–2013*	*2014–2017*	*2011–2013*	*2014–2017*	
	*(*n *= 4,973)*	*(*n *=* *7,394)*	*(*n *=* *8,789)*	*(*n *=* *13,790)*		*(*n *=* *17,190)*	*(*n *=* *20,066)*	*(*n *=* *38,931)*	*(*n *=* *53,252)*	
Uninsured										
Non-Hispanic white	36.4	10.1	35.8	39.0	− 29.46 (− 33.95 to − 24.96)	18.4	6.6	20.9	20.5	− 11.50 (− 12.59 to − 10.41)
Non-Hispanic black	42.1	10.9	50.8	51.5	− 31.88 (− 34.36 to − 29.40)	24.9	8.2	27.0	26.7	− 16.42 (− 18.47 to − 14.37)*
Hispanic	40.6	21.3	47.3	45.5	− 17.50 (− 24.78 to − 10.22)*	31.1	18.4	26.1	25.7	− 12.25 (− 15.47 to − 9.04)
Medicaid										
Non-Hispanic white	18.0	50.2	16.6	15.9	32.93 (28.65 to 37.21)	11.2	27.6	10.2	9.7	16.93 (15.83 to 18.04)
Non-Hispanic black	25.9	65.4	17.9	19.5	38.00 (35.67 to 40.35)*	12.8	24.8	13.7	13.0	23.07 (20.84 to 25.29)*
Hispanic	14.6	49.2	19.4	22.9	31.13 (24.67 to 37.60)	12.8	24.8	10.0	12.0	10.03 (7.32 to 12.75)*
Private insurance										
Non-Hispanic white	33.9	30.4	28.6	28.8	− 3.73 (− 8.48 to − 1.03)	59.5	53.9	56.1	58.8	− 8.24 (− 9.76 to − 6.72)
Non-Hispanic black	18.8	18.9	16.6	15.7	1.00 (− 1.08 to 3.08)	45.1	39.7	41.4	44.8	− 8.85 (− 11.44 to − 6.26)
Hispanic	33.7	23.3	15.8	16.1	− 10.73 (− 17.22 to − 4.24)	48.3	47.3	56.0	53.9	1.14 (− 2.56 to 4.85)*
In-hospital mortality										
Non-Hispanic white	10.8	9.6	11.2	11.1	− 1.13 (− 0.37 to 1.43)	2.5	2.1	2.4	2.3	− 0.21 (− 0.64 to 0.22)
Non-Hispanic black	7.7	5.3	5.0	4.4	− 1.84 (− 0.29 to − 0.75)	2.3	2.3	2.2	1.9	0.26 (− 0.46 to 0.98)
Hispanic	5.4	5.5	6.0	6.3	− 0.23 (− 3.27 to 2.82)	2.2	1.4	2.5	2.1	− 0.40 (− 1.37 to 0.56)
Discharged to any rehabilitation										
Non-Hispanic white	17.1	16.6	15.8	14.2	1.15 (− 2.93 to 5.22)	21.2	23.5	21.6	22.7	1.24 (− 0.10 to 2.59)
Non-Hispanic black	14.3	16.7	12.1	11.7	2.82 (0.72 to 4.93)	21.8	25.7	20.7	20.8	3.85 (1.20 to 6.49)*
Hispanic	18.6	17.4	13.0	12.2	− 0.41 (− 6.03 to 5.22)	20.3	21.7	16.6	18.1	− 0.09 (− 3.18 to 3.00)
Discharged to inpatient rehabilitation										
Non-Hispanic white	4.6	6.2	4.4	5.0	1.03 (− 1.29 to 3.34)	10.6	13.0	10.5	11.5	1.36 (0.35 to 2.37)
Non-Hispanic black	4.0	6.1	4.6	4.6	2.06 (0.81 to 3.30)	9.1	11.5	9.1	10.1	1.34 (− 0.61 to 3.28)
Hispanic	5.2	5.4	5.4	5.0	0.50 (− 2.79 to 3.80)	6.6	8.7	8.0	9.0	1.04 (− 0.99 to 3.06)
Discharged to a skilled nursing facility										
Non-Hispanic white	0.6	0.8	1.0	0.8	0.35 (− 0.60 to 1.31)	2.1	2.4	2.1	2.0	0.46 (− 0.02 to 0.95)
Non-Hispanic black	0.9	0.8	0.8	0.6	0.09 (− 0.44 to 0.62)	1.9	3.5	2.5	2.2	1.96 (0.94 to 2.99)*
Hispanic	0.4	0.2	1.0	0.4	0.38 (− 0.71 to 1.47)	1.9	2.5	1.4	1.3	0.73 (− 0.36 to 1.82)
Discharged to home health care										
Non-Hispanic white	12.0	9.6	10.4	8.4	− 0.31 (− 3.82 to 3.21)	8.6	8.1	9.0	9.2	− 0.63 (− 1.56 to 0.30)
Non-Hispanic black	9.5	9.8	6.8	6.5	0.61 (− 1.17 to 2.39)	10.9	10.7	9.1	8.4	0.44 (− 1.63 to 2.51)
Hispanic	13.2	12.2	6.5	6.7	− 1.29 (− 6.20 to 3.62)	11.7	10.7	7.3	7.8	− 1.61 (− 4.04 to 0.82)
30-d unplanned readmission										
Non-Hispanic white	10.7	11.3	9.5	11.8	− 1.68 (− 7.67 to 4.30)	8.6	7.6	7.9	7.9	− 0.91 (− 2.45 to 0.62)
Non-Hispanic black	9.2	9.9	8.9	8.7	0.89 (− 1.87 to 3.64)	8.2	9.8	6.5	7.2	0.99 (− 1.36 to 3.34)
Hispanic	7.9	8.4	11.4	8.8	3.07 (− 5.01 to 11.14)	3.7	6.1	6.5	6.8	2.13 (− 0.50 to 4.76)
30-d return ED visit										
Non-Hispanic white	16.9	23.1	19.5	22.5	3.18 (− 4.55 to 10.90)	16.3	15.8	18.2	18.5	− 0.81 (− 2.92 to 1.30)
Non-Hispanic black	21.7	25.3	25.6	25.8	3.39 (− 0.70 to 7.49)	14.8	18.6	18.7	18.9	3.51 (0.21 to 6.81)*
Hispanic	24.2	22.5	19.4	22.6	− 4.88 (− 16.47 to 6.71)	14.7	14.4	16.9	16.8	− 0.12 (− 4.59 to 4.35)

Risk-adjusted marginal percentages are shown. *P < .05 versus difference-in-difference in non-Hispanic white patients.

With regard to the differential impact of Medicaid expansion by a patient's community income quartile, the Medicaid expansion–associated increase in the proportion of MVC trauma patients discharged to inpatient rehabilitation was larger among patients from lower- and moderate-income communities than among patients from the highest-income communities (Table 4). There were no significant differences in the impact of Medicaid expansion on any of the other evaluated trauma outcomes by community income among either MVC or firearm trauma patients.

**Table 4:** Association of the ACA Medicaid expansion with insurance coverage and risk-adjusted outcomes among young adult firearm and MVC trauma patients: results by community income level

	*Firearm trauma patients*					*MVC trauma patients*				
	*Expansion states*		*Nonexpansion states*		*Difference-in-differences estimate (95% CI)*	*Expansion states*		*Nonexpansion states*		*Difference-in-differences estimate (95% CI)*
	*2011–2013*	*2014–2017*	*2011–2013*	*2014–2017*		*2011–2013*	*2014–2017*	*2011–2013*	*2014–2017*	
	*(*n *= 4,973)*	*(*n *= 7,394)*	*(*n *= 8,789)*	*(*n *= 13,790)*		*(*n *= 17,190)*	*(*n *= 20,066)*	*(*n *= 38,931)*	*(*n *= 53,252)*	
Uninsured										
Quartile 1 (lowest)	43.2	12.2	51.5	51.3	− 30.85 (− 33.59 to − 28.12)	27.5	8.3	25.6	25.4	− 18.97 (− 20.65 to − 17.29)*
Quartile 2	40.7	12.4	42.9	44.8	− 30.17 (− 34.69 to − 25.66)	24.3	9.7	22.6	22.8	− 14.72 (− 16.70 to − 12.74)*
Quartile 3	38.5	10.5	40.0	41.0	− 28.97 (− 34.27 to − 23.68)	19.5	8.5	20.4	19.2	− 9.82 (− 11.64 to − 8.00)*
Quartile 4 (highest)	32.1	9.5	34.3	38.4	− 26.74 (− 34.85 to − 18.63)	13.3	7.1	19.2	16.6	− 3.58 (− 5.72 to − 1.44)
Medicaid										
Quartile 1 (lowest)	23.2	64.0	18.9	20.3	39.48 (36.94 to 42.02)*	17.2	39.2	13.8	13.0	22.81 (21.07 to 24.55)*
Quartile 2	22.0	60.2	17.2	18.5	36.87 (32.55 to 41.19)	13.0	29.7	11.5	10.5	17.80 (15.85 to 19.74)*
Quartile 3	24.6	54.9	15.0	15.3	29.97 (25.04 to 34.90)	13.4	27.5	7.7	8.1	13.72 (11.92 to 15.51)
Quartile 4 (highest)	23.6	50.8	14.7	10.7	31.18 (23.94 to 38.42)	9.8	22.5	5.5	6.0	12.17 (10.33 to 14.01)
Private insurance										
Quartile 1 (lowest)	20.3	18.8	14.8	14.9	− 1.64 (− 3.92 to 0.63)	41.7	40.7	46.7	49.8	− 4.17 (− 6.27 to − 2.07)
Quartile 2	23.4	21.6	22.0	21.3	− 1.12 (− 5.31 to 3.06)	52.3	49.3	53.5	55.4	− 4.90 (− 7.46 to − 2.34)
Quartile 3	28.2	27.4	28.2	28.5	− 2.95 (− 5.60 to − 0.30)	58.8	53.3	58.5	60.1	− 7.00 (− 9.48 to − 4.53)
Quartile 4 (highest)	32.1	32.0	34.3	37.6	− 1.49 (− 5.62 to 2.63)	70.4	61.0	61.7	65.1	− 12.70 (− 15.64 to − 9.76)
In-hospital mortality										
Quartile 1 (lowest)	8.2	6.1	6.1	5.4	− 1.36 (− 2.61 to − 0.12)	1.9	2.3	2.3	2.2	0.48 (− 0.09 to 1.05)
Quartile 2	7.4	6.7	7.0	7.1	− 0.85 (− 2.98 to 1.28)	3.1	2.0	2.4	2.1	− 0.68 (− 1.42 to 0.06)
Quartile 3	9.9	7.1	7.0	7.1	0.02 (− 0.35 to 0.39)	2.3	2.4	2.6	2.5	0.27 (− 0.44 to 0.98)
Quartile 4 (highest)	9.2	7.3	8.6	8.3	− 0.21 (− 0.64 to 0.23)	2.7	1.8	2.3	2.1	− 0.69 (− 1.53 to 0.14)
Discharged to any rehabilitation										
Quartile 1 (lowest)	13.2	15.4	11.9	11.6	2.55 (0.39 to 4.70)	18.5	22.1	19.8	20.1	3.31 (1.59 to 5.04)
Quartile 2	17.9	16.7	14.8	12.3	1.33 (− 2.67 to 5.34)	20.6	23.1	21.1	22.1	1.48 (− 0.70 to 3.65)
Quartile 3	19.9	20.8	13.1	14.6	− 0.62 (− 5.79 to 4.56)	22.4	25.6	21.1	22.2	2.01 (− 0.31 to 4.32)
Quartile 4 (highest)	19.2	18.8	19.2	13.9	4.89 (− 3.44 to 13.21)	26.7	26.5	23.0	24.1	− 1.33 (− 4.50 to 1.83)

Discharged to inpatient rehabilitation										
Quartile 1 (lowest)	4.0	6.0	4.6	4.8	1.81 (0.51 to 3.12)	8.6	11.7	9.1	9.9	2.34 (1.08 to 3.60)*
Quartile 2	4.9	5.4	5.0	4.4	1.05 (− 1.19 to 3.29)	10.0	12.2	9.7	11.2	0.70 (− 0.90 to 2.30)*
Quartile 3	5.2	7.1	3.9	5.7	0.10 (− 2.85 to 3.05)	10.5	12.7	10.9	11.0	2.06 (0.36 to 3.77)*
Quartile 4 (highest)	4.4	6.1	6.7	4.0	4.31 (− 0.33 to 8.95)	12.1	12.3	10.8	13.3	− 2.18 (− 4.51 to 0.16)
Discharged to a skilled nursing facility										
Quartile 1 (lowest)	0.8	0.8	0.7	0.6	0.09 (− 0.46 to 0.65)	1.3	1.9	2.2	2	0.73 (0.16 to 1.30)
Quartile 2	0.7	0.3	1.1	0.6	0.10 (− 0.75 to 0.96)	2.2	2.7	2.2	1.8	0.86 (0.03 to 1.70)
Quartile 3	0.4	1.4	0.6	0.5	1.00 (− 0.15 to 2.15)	2.4	3.1	2.0	2.1	0.65 (− 0.24 to 1.54)
Quartile 4 (highest)	1.0	0.3	1.4	0.7	0.00 (− 2.04 to 2.04)	2.6	3.8	2.0	1.7	1.42 (0.23 to 2.60)
Discharged to home health care										
Quartile 1 (lowest)	8.5	8.7	6.6	6.2	0.60 (− 1.18 to 2.37)	8.6	8.5	8.6	8.2	0.20 (− 1.05 to 1.45)
Quartile 2	12.3	11.1	8.7	7.3	0.13 (− 3.38 to 3.65)	8.5	8.1	9.2	9.0	− 0.22 (− 1.75 to 1.31)
Quartile 3	14.4	12.5	8.6	8.4	− 1.75 (− 6.31 to 2.80)	9.6	9.7	8.3	9.1	− 0.70 (− 2.35 to 0.95)
Quartile 4 (highest)	14.3	12.4	11.1	9.2	0.00 (− 7.42 to 7.43)	12.0	10.3	10.1	9.1	− 0.64 (− 2.96 to 1.67)
30-d unplanned readmission										
Quartile 1 (lowest)	7.8	9.2	8.4	9.2	0.53 (− 2.48 to 3.54)	7.3	6.9	7.0	7.1	− 0.54 (− 2.72 to 1.65)
Quartile 2	12.0	10.4	10.1	9.1	− 0.57 (− 6.02 to 4.87)	6.2	7.9	6.3	6.4	1.57 (− 0.77 to 3.91)
Quartile 3	8.9	11.7	9.2	9.1	2.85 (− 3.29 to 8.99)	8.2	7.9	7.4	7.9	− 0.74 (− 3.09 to 1.61)
Quartile 4 (highest)	13.3	12.5	12.3	12.0	− 0.52 (− 12.22 to 11.19)	11.2	11.1	10.8	10.2	0.46 (− 3.18 to 4.11)
30-d return ED visit										
Quartile 1 (lowest)	22.8	25.3	24.7	25.6	1.60 (− 3.06 to 6.26)	17.1	18.7	19.2	20.3	0.53 (− 2.58 to 3.63)
Quartile 2	19.6	24.5	23.9	25.6	3.21 (− 4.00 to 10.42)	16.1	16.0	18.8	18.7	− 0.02 (− 3.39 to 3.34)
Quartile 3	16.3	23.2	20.8	21.4	6.27 (− 2.01 to 14.55)	15.0	15.3	16.6	16.6	0.28 (− 2.84 to 3.40)
Quartile 4 (highest)	20.8	20.4	16.4	17.5	− 1.54 (− 15.12 to 12.04)	11.3	12.3	13.9	14.2	0.69 (− 3.27 to 4.65)

Risk-adjusted marginal percentages are shown. *P < `.05 vs. difference-in-difference in patients from the highest community income quartile.

In the subset of trauma patients with 1 or more of the diagnoses defined by CMS as a requirement for financial support of such a facility (ie, the 60% rule), the proportion of firearm trauma patients who were discharged to inpatient rehabilitation increased significantly more in Medicaid expansion states than in nonexpansion states (Supplemental Table 2). However, this increase did not significantly differ by patient race/ethnicity or community income level. Among MVC trauma patients meeting the CMS criteria, the proportion of patients who were discharged to inpatient rehabilitation increased significantly in both Medicaid expansion and nonexpansion states, and the DD estimate was not statistically significant, although it was similar in magnitude to that seen in the overall MVC trauma patient cohort (Supplemental Table 3). However, Medicaid expansion was associated with a significant decrease in the socioeconomic disparity in the proportion of young adult MVC trauma patients with these presumptive injury diagnoses who were discharged to inpatient rehabilitation, with patients from the lowest-income communities benefiting the most from Medicaid expansion.

## DISCUSSION

Among young adults hospitalized for firearm- or MVC-related injuries, this study found that the first 4 years of the ACA Medicaid expansion were associated with decreases in the proportion of patients who were uninsured, increases in the proportion of patients covered by Medicaid, and increases in the proportion of patients discharged to any type of rehabilitative care and to specifically inpatient rehabilitation. Medicaid expansion was also associated with a significant decrease in the proportion of firearm trauma patients who died in the hospital, but no such association was seen among MVC trauma patients. In both subsets of the young adult trauma patient population, there were no Medicaid expansion–associated changes in 30-day unplanned readmissions or return ED visits. Increases in Medicaid coverage were larger among black than white patients among both firearm and MVC trauma patients. In contrast, the decrease in the uninsured rate was smaller among Hispanic than white or black firearm trauma patients, and the increase in Medicaid coverage was smaller among Hispanic than white or black firearm trauma patients. Among MVC trauma patients, those patients from lower-income communities experienced larger increases in insurance coverage overall and Medicaid coverage than patients from higher-income communities. Among firearm trauma patients, gains in insurance coverage were similar across groups defined by community income quartile. Additionally, among firearm trauma patients, the associations of Medicaid expansion with all evaluated trauma outcomes and measures of access to rehabilitative care were also similar by patient race/ethnicity and community income quartile. Among MVC trauma patients, black patients compared to white patients experienced larger Medicaid expansion–associated increases in discharge to any rehabilitation, discharge to skilled nursing, and 30-day return ED visits. Lastly, MVC patients from lower-income communities experienced larger Medicaid expansion–associated increases in the proportion of patients discharged to inpatient rehabilitation than patients from high-income communities. Overall, these results suggest that Medicaid expansion has had generally positive effects on insurance coverage, in-hospital mortality, and access to rehabilitative care among young adult firearm trauma patients. It has also had positive effects on insurance coverage and access to rehabilitative care and has reduced some racial and socioeconomic disparities in these outcomes among young adult MVC trauma patients.

The rise in Medicaid coverage following the ACA Medicaid expansion was clearly the primary driver of increased insurance coverage among trauma patients in the expansion states. Among both firearm and MVC trauma patients, the overall insurance coverage rate and Medicaid coverage rate rose sharply during the first 2 years following Medicaid expansion, whereas private insurance either dropped or remained stable. The uninsured rate by the year 2017 was only 11.7% among firearm trauma patients and 8.4% among MVC trauma patients in the 5 selected Medicaid expansion states. Correspondingly, in the 5 nonexpansion states, the uninsured rate was 48.0% and 22.8% among firearm and MVC trauma patients, respectively, in 2017. Nevertheless, racial/ethnic disparities in insurance coverage rates among young adult firearm and MVC trauma patients persisted in 2017, with Hispanic patients still being more likely to be uninsured than either black or white patients after Medicaid expansion. This may be partly due to Hispanic patients being disproportionately affected by the current policy that makes illegal immigrants and legal permanent residents who have resided in the United States for less than 5 years ineligible for Medicaid [48]. Language barriers as well as public charge rule changes and other immigration-related policy changes in recent years likely also played a role [[49], [50], [51]]. One interesting finding of this study was that Medicaid expansion led to greater decreases in the uninsured rate among patients from lower- and moderate-income communities among MVC trauma patients but decreases in the uninsured rate were similar across groups defined by community-level income among firearm trauma patients. One potential reason for this is that firearm trauma patients across all community income levels were much more likely to be uninsured than MVC trauma patients before the ACA. Furthermore, regardless of the SES of a community, its most socioeconomically vulnerable residents tend to be at higher risk of firearm injury [14,52].

Among both firearm and MVC trauma patients, Medicaid expansion was associated with increases in discharge to any rehabilitation and to inpatient rehabilitation. Among firearm trauma patients, the increase in access to inpatient rehabilitation was particularly large among patients who had 1 or more of the types of injuries meeting CMS criteria for inpatient rehabilitation, such as spinal cord injury or severe brain injury. Before Medicaid expansion, the high rates of uninsurance among MVC and firearm trauma patients limited physicians' ability to refer these patients for appropriate postacute care. Medicaid expansion has thus directly led to increased access to appropriate rehabilitative care, particularly among firearm trauma patients with the types of injuries that would most benefit from inpatient rehabilitation [27]. This has likely improved the long-term function and quality of life of these patients, but additional research evaluating this is needed. Interestingly, these improvements in access to rehabilitation among firearm trauma patients did not differ by patient race/ethnicity or community income quartile. This is likely because there were only minimal differences in these outcomes across these groups in the selected Medicaid expansion states prior to Medicaid expansion. On the other hand, among MVC trauma patients, black patients experienced a greater increase in discharge to any rehabilitation, discharge to skilled nursing facilities, and return ED visits within 30 days when compared to white patients. This is likely because, among MVC trauma patients, black patients were more likely than white patients to be uninsured before Medicaid expansion. In addition, among patients with MVC-related injuries, the proportion of patients from low-income communities was much higher among black patients than white patients. As a result of their larger gains in coverage, more black patients and patients from-lower income communities were able to access rehabilitation.

This study found no association of Medicaid expansion with rates of 30-day unplanned readmissions or return ED visits among either young adult firearm or MVC trauma patients. Our previous analyses in the overall young adult trauma population found similar results [26,30]. Although we expected Medicaid expansion to be associated with reduced use of ED care, studies evaluating this in the general adult population or in specific subgroups have yielded mixed results [53]. The greater availability of ED care in expansion states as compared to nonexpansion states may partly explain the lack of an association of Medicaid expansion with return ED visits in this study [54]. The greater reliance, on average, of black patients than white patients on ED care may explain our finding of an association between Medicaid expansion and greater return ED visit rates among black patients with MVC-related injuries [55].

Previous research has shown that uninsured trauma patients are at higher risk for in-hospital mortality even after accounting for confounding demographic, clinical, and injury characteristics [3,4,[6], [7], [8]]. Our investigation found Medicaid expansion to be associated with decreased in-hospital mortality among firearm trauma patients, with in-hospital mortality decreasing by 1.5 percentage points more in Medicaid expansion than nonexpansion states. From data available in the American Hospital Association's (AHA) Annual Survey Database on the hospitals in states whose SIDs had AHA hospital identifiers, we determined that there was a small but statistically significant increase in the percentage of firearm trauma patients treated at level 1 or 2 trauma centers post–Medicaid expansion in the 5 selected Medicaid expansion states (88.6% vs 86.6%, *P* < .001) but no such increase in the 2 nonexpansion states, namely, Florida and North Carolina, for which such data were available (82.1% to 81.8%; *P* = .60) (data not shown). Therefore, it is possible that some firearm trauma patients received higher-quality care at better equipped hospitals after Medicaid expansion, which may have contributed to the Medicaid expansion–associated decrease in in-hospital mortality. However, without information on hospital characteristics for most patients treated in the nonexpansion states, we are unable to confirm this. Alternatively, the Medicaid expansion–associated decrease in in-hospital mortality among firearm trauma patients could also have resulted from improved access to high-quality prehospital care, more timely access to definitive care, or higher-quality hospital care regardless of hospital trauma center status. Unfortunately, the SIDs do not provide the data necessary to evaluate the relative importance of these potential mechanisms. Interestingly, we did not observe a similar Medicaid expansion–associated decrease in in-hospital mortality among young adult MVC trauma patients. Previous studies evaluating the impact of the ACA Medicaid expansion on nonelderly adult trauma patients have mostly found Medicaid expansion to be unassociated with changes in in-hospital mortality, although 1 study of Maryland trauma patients in the first 2 years of Medicaid expansion did find a decrease, and our previous study evaluating all young adult trauma patients in the same 10 states examined in the present study did find a decrease among specifically black trauma patients [26,28]. Numerous factors affect in-hospital mortality risk, and many of these factors are not available in administrative hospital discharge databases, which make it difficult to isolate the effects of Medicaid expansion on this outcome [[56], [57], [58]]. The in-hospital mortality rate is also quite low among young adult trauma patients, particularly among trauma patients not injured by firearms.

Although this study sheds substantial light on the impact of Medicaid expansion on young adults hospitalized for firearm- or MVC-related injuries, it has some limitations. First, there is the possibility that some patients' injuries or other characteristics were misclassified, as the SIDs do not contain detailed clinical information. The concern about data misclassification is perhaps magnified by the fact that the study period overlaps with the ICD-9-CM to ICD-10-CM coding system transition. To minimize the effects of this transition, we previously conducted a study that examined its impact on trends in traumatic injury–related hospitalizations among young adults by injury mechanism, type, and severity, and the results of that study informed our selection and definition of covariates in the present study [41]. The prevalence of many injury types was affected by the coding transition. Thus, to minimize any bias that would result from this, we elected to not adjust for granular injury types. However, we acknowledge that this likely led to some unmeasured confounding. Second, our study investigated only 10 states. Although we attempted to select states with comparable geographic, patient sociodemographic, and trauma system characteristics, there likely remain some pre-ACA differences between our Medicaid expansion and nonexpansion cohorts. Moving forward, additional studies should evaluate Medicaid expansion's effects on firearm and MVC trauma patients' long-term health and functional, social, and economic outcomes.

In conclusion, this study was the first large population-based study to evaluate the effects of the ACA Medicaid Expansion on young adults hospitalized for firearm- or MVC-related injuries. As expected, the Medicaid expansion–associated decrease in the uninsured rate was greater among young adult firearm trauma patients than MVC trauma patients. Nevertheless, both groups experienced similar Medicaid expansion–associated increases in access to rehabilitation of approximately 2 percentage points during 2014–2017. Among firearm trauma patients, Medicaid expansion was also associated with a reduction in in-hospital mortality of approximately 1.5 percentage points. Considering the confidence interval around this estimate, Medicaid expansion in its first 4 years appears to have led to between 6 and 25 fewer in-hospital deaths per 1,000 hospitalized young adult firearm trauma patients. Medicaid expansion has likely also led to improvements in trauma patients' economic stability and long-term quality of life as well as reductions in racial and socioeconomic disparities in these outcomes. However, future studies examining the long-term impact of the ACA Medicaid expansion on trauma patients are warranted.

## Author Contributions

JNC and LA conceived of and designed the study. All authors were responsible for the acquisition, analysis, and interpretation of the data. MRR, PMH, and JNC drafted the manuscript. All authors made critical revisions to the manuscript.

## Conflicts of Interest

None.

## Funding Sources

This work was supported in part by the Ohio State College of Medicine Samuel J. Roessler Memorial Scholarship (MR). This work was also supported by the National Institute on Minority Health and Health Disparities of the 10.13039/100000002National Institutes of Health [Award Number R01MD013881] (JNC). Neither sponsor had any role in study design; in the collection, analysis, or interpretation of data; in the writing of the report; or in the decision to submit the article for publication. The content is solely the responsibility of the authors and does not necessarily represent the official views of the National Institutes of Health. The authors have no other disclosures to report.

## Ethics Approval

This study was approved by the Institutional Review Board of the Illinois Department of Public Health and by the Nationwide Children's Hospital Institutional Review Board with a waiver of informed consent.

## Appendix A Supplementary data

Supplementary tables Image 1
