# Supplementary material for: Impact of Medicaid expansion on young adult firearm and motor vehicle crash trauma patients
Source: Surg Open Sci. 2022 Feb 1;8:9–19. doi: 10.1016/j.sopen.2022.01.004 (PMC8881723; doi:10.1016/j.sopen.2022.01.004)
Supplement: Supplementary file 1 — Supplementary tables [file mmc1.docx]

**Supplemental Tables**

Supplemental Table S1. Population and trauma system characteristics of the five selected Medicaid expansion and five selected non-expansion states in 2013

|  | **Expansion States** | **Non-expansion States** |
| --- | --- | --- |
| **Characteristics of residents aged 18-44 years** |  |  |
| Uninsured | 21% | 28% |
| Non-Hispanic Black or Hispanic | 31% | 38% |
| Income below federal poverty level | 17% | 18% |
| Urban/rural residence |  |  |
| Large central metropolitan (>1 million population) | 27% | 25% |
| Large fringe metropolitan (>1 million population) | 35% | 29% |
| Medium metropolitan (250,000-999,999 population) | 17% | 22% |
| Small metropolitan (50,000-249,999 population) | 11% | 10% |
| Micropolitan/noncore (rural) | 20% | 15% |
| Bachelor’s degree or higher | 37% | 30% |
| **Trauma system characteristics** |  |  |
| Residents within 60 minutes of level I or II trauma center | 86% | 93% |
| Level I or II trauma centers per 1 million residents | 1.9 | 1.2 |
| Emergency departments per 1 million residents | 17.0 | 13.0 |
| CDC 2011 field triage guidelines used | 3 of 5 states | 2 of 5 states |
| Trauma destination bypass protocols in place | 4 of 5 states | 4 of 5 states |
| Data sources:  1. United States Census Bureau. American FactFinder. 2013 American Community Survey 1-Year Estimates.  https://factfinder.census.gov. Accessed 2/8/2018.  2. America's Emergency Care Environment. A State-by-State Report Card-2014.  http://www.emreportcard.org/. Accessed 4/18/2018.  3. National Association of State EMS Officials. Status of State Trauma System Planning and Development. Falls Church, VA September 2016. https://www.nasemso.org/Resources/Monographs/documents/Status-of-State-Trauma-System-Planning-and-Development-Sept2016.pdf. Accessed September 2016. | | |

Supplemental Table S2. Rates of discharge to inpatient rehabilitation among young adult firearm trauma patients with injuries meeting the Centers for Medicare & Medicaid Services’ criteria for inpatient rehabilitation in five Medicaid expansion and five non-expansion states

|  | **Expansion States** | | **Non-expansion States** | | **Difference-in-Differences Estimate (95% CI)** | **P** |
| --- | --- | --- | --- | --- | --- | --- |
|  | **2011-2013** | **2014-2017** | **2011-2013** | **2014-2017** |  |  |
|  | (n=956) | (n=1,549) | (n=1,562) | (n=2,530) |  |  |
| Overall | 21.9 | 26.4* | 24.9 | 23.8 | 5.62 (0.67 to 10.57) | **0.03** |
| **By race/ethnicity** | | | | | | |
| Non-Hispanic White | 22.6 | 26.2 | 23.4 | 20.4 | 6.60 (-5.01 to 18.22) | 0.27 |
| Non-Hispanic Black | 20.7 | 27.2 | 25.3 | 24.7 | 7.11 (1.05 to 13.16) | 0.02 |
| Hispanic | 24.2 | 23.4 | 24.1 | 24.7 | -1.47 (-16.75 to 13.82) | 0.85 |
| **By community-level income quartile** | | | | | | |
| Quartile 1 (lowest) | 21.5 | 27.0 | 24.9 | 24.7 | 5.71 (-0.77 to 12.20) | 0.08 |
| Quartile 2 | 25.3 | 24.4 | 25.2 | 20.0 | 4.34 (-6.52 to 15.21) | 0.43 |
| Quartile 3 | 18.9 | 26.1 | 22.2 | 28.7 | 0.74 (-12.34 to 13.82) | 0.91 |
| Quartile 4 (highest) | 22.2 | 29.1 | 29.6 | 22.3 | 14.22 (-7.90 to 36.34) | 0.21 |

Risk-adjusted marginal percentages are shown. *p<0.05 vs. years 2011-2013 in the same states.

Supplemental Table S3. Rates of discharge to inpatient rehabilitation among young adult motor vehicle crash trauma patients with injuries meeting the Centers for Medicare & Medicaid Services’ criteria for inpatient rehabilitation in five Medicaid expansion and five non-expansion states

|  | **Expansion States** | | **Non-expansion States** | | **Difference-in-Differences Estimate (95% CI)** | **P** |
| --- | --- | --- | --- | --- | --- | --- |
|  | **2011-2013** | **2014-2017** | **2011-2013** | **2014-2017** |  |  |
|  | (n=7,011) | (n=8,756) | (n=15,775) | (n=22,532) |  |  |
| Overall | 18.7 | 22.0* | 18.3 | 20.3* | 1.41 (-0.23 to 3.05) | 0.09 |
| **By race/ethnicity** | | | | | | |
| Non-Hispanic White | 20.2 | 24.0 | 19.7 | 21.6 | 1.93 (-0.16 to 4.02) | 0.07 |
| Non-Hispanic Black | 17.5 | 20.6 | 16.8 | 18.6 | 1.25 (-2.71 to 5.20) | 0.54 |
| Hispanic | 12.4 | 16.3 | 14.7 | 17.2 | 1.37 (-2.88 to 5.62) | 0.53 |
| **By community-level income quartile** | | | | | | |
| Quartile 1 (lowest) | 16.8 | 21.7 | 17.1 | 18.4 | 3.66 (1.07 to 6.25)** | **0.006** |
| Quartile 2 | 19.0 | 21.2 | 18.3 | 21.0 | -0.51 (-3.81 to 2.79) | 0.76 |
| Quartile 3 | 20.1 | 23.1 | 19.9 | 20.8 | 1.97 (-1.59 to 5.53) | 0.20 |
| Quartile 4 (highest) | 21.9 | 22.4 | 20.1 | 24.8 | -4.19 (-8.94 to 0.56) | 0.08 |

Risk-adjusted marginal percentages are shown. *p<0.05 vs. years 2011-2013 in the same states. **difference-in-difference estimate p<0.05 vs. highest community income quantile group
